# Comparison of urine proteome among rat models by intraperitoneal injection with single bacteria and co-injection with two bacteria

**DOI:** 10.1371/journal.pone.0261488

**Published:** 2021-12-31

**Authors:** Wenshu Meng, Chenyang Zhao, Youhe Gao

**Affiliations:** Gene Engineering Drug and Biotechnology Beijing Key Laboratory, College of Life Sciences, Beijing Normal University, Beijing, China; Aarhus University, DENMARK

## Abstract

**Purpose:**

To explore and compare urine proteome changes among rat models by intraperitoneal injection with single bacteria and co-injection with two bacteria.

**Method:**

*Escherichia coli* and *Staphylococcus aureus* are two common human pathogens. Three rat models were established: (i) the intraperitoneal co-injection of *E*. *coli* and *S*. *aureus* model (ES model), (ii) intraperitoneal injection of *E*. *coli* model (E model), and (iii) intraperitoneal injection of *S*. *aureus* model (S model). Urinary proteomes on days 0, 1 and 2 of the three models were analyzed by liquid chromatography coupled with tandem mass spectrometry (LC-MS/MS).

**Results:**

A total of 111, 34 and 94 differential proteins were identified in the ES model, E model and S model, respectively. Among them, some differential proteins were reported to be associated with bacterial infection. Approximately 47% differential proteins in the E model overlapped with ES model, and 37% differential proteins in the S model overlapped with ES model. Compared with the E model and S model, a total of 71 unique differential proteins were identified in the ES model.

**Conclusion:**

Our results indicated that (1) the urine proteome could distinguish different bacterial intraperitoneal injections models and (2) the effects of co-injection with two bacteria on the urine proteome were not simple superposition of single injection.

## Introduction

*Escherichia coli (E*. *coil)* is a gram-negative bacteria and *Staphylococcus aureus (S*. *aureus)* is a gram-positive bacteria, which are the two common human pathogens and cause a wide range of clinical infections [1, 2]. Bacterial coinfections are common in many diseases [3–5]. Differential diagnosis of many bacterial infections remains difficult.

Urine is a good source for disease diagnostic biomarkers [6]. Whether biomarkers for multifactorial complex diseases are superposition of biomarkers for individual factor is unclear. Recent studies showed that urinary proteomics could reflect changes of infection-related diseases, such as bacterial meningitis [7] and *T*. *gondii* infection [8]. However, whether the urine proteome can distinguish between single infections and coinfections of two bacteria is unknown.

**Fig 1:**
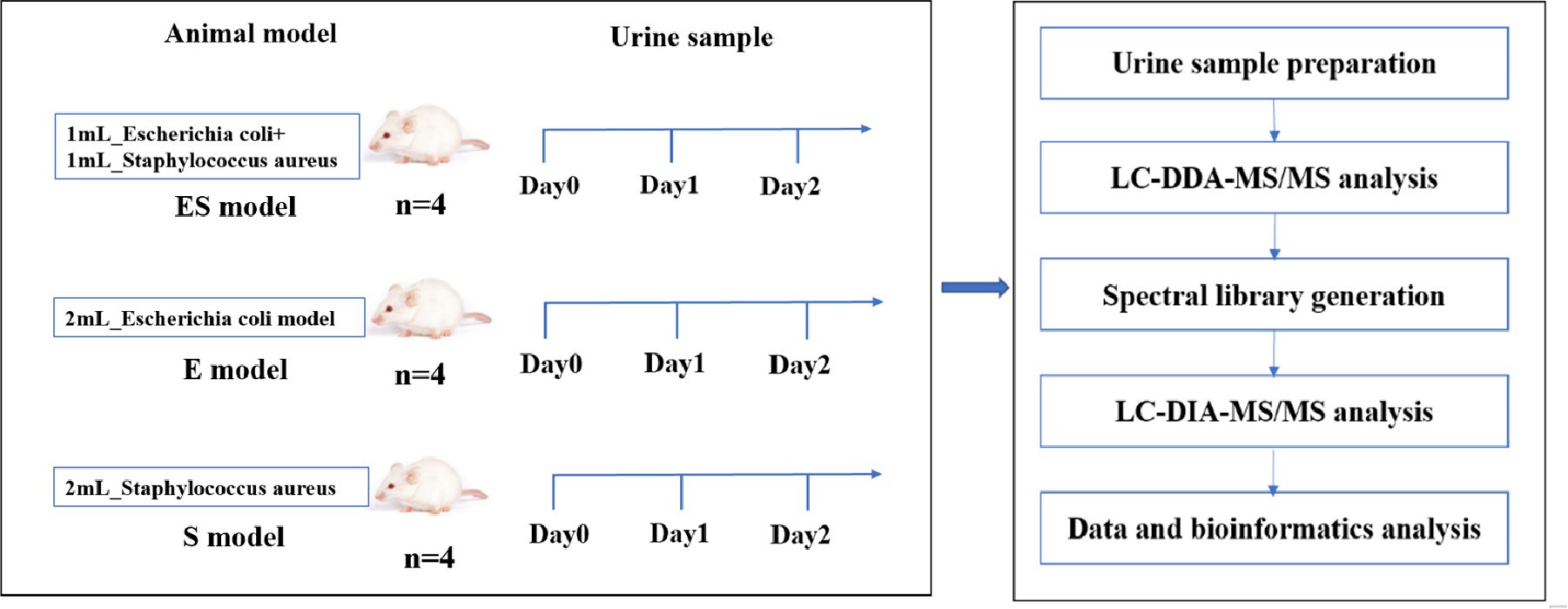
The workflow of this study.

In this study, we established three rat models: (i) the intraperitoneal co-injection of *E*. *coli* and *S*. *aureus* model (ES model), (ii) intraperitoneal injection of *E*. *coli* model (E model), and (iii) intraperitoneal injection of *S*. *aureus* model (S model). The urinary proteomes of the three models were analyzed by liquid chromatography coupled with tandem mass spectrometry (LC-MS/MS). The purpose of this study was to explore and compare urine proteome changes among two bacterial single injection and co-injection rat models. The workflow of this study is shown in Fig 1.

## Materials & methods

### Experimental animals and model establishment

Male Wistar rats (n = 12, 190 ± 20 g) were purchased from Beijing Vital River Laboratory Animal Technology Co., Ltd. Animals were fed a standard laboratory diet under controlled indoor temperature (21 ± 2°C), humidity (65–70%) and 12 h/12 h light-dark cycle conditions. The study was approved by Peking Union Medical College (Approval ID: ACUC-A02-2014-007) and performed according to the guidelines developed by the Institutional Animal Care and Use Committee. After the experiment, all the animals were euthanized by intraperitoneal injection of barbiturates.

*Escherichia coli* and *Staphylococcus aureus* were obtained from the Department of Biochemistry and Molecular Biology, School of Life Sciences, Beijing Normal University (Beijing, China) and were intraperitoneally injected into Wistar rats. The concentration of bacteria was evaluated by using UV-VIS spectrophotometer to measure the absorbance at 600 nm. *E*. *coli* and *S*. *aureus* suspensions were diluted to a concentration of 1×10^9^ CFU/ml with normal saline (NS) [9]. After three days of acclimatization, the rats were randomly divided into the following two groups: the control group (n = 4) and the experimental group (n = 12). In the experimental group, rats were intraperitoneally injected with 1 ml *E*. *coli* and 1 ml *S*. *aureus* (n = 4), rats were intraperitoneally injected with 2 ml *E*. *coli* (n = 4), or rats were intraperitoneally injected with 2 ml *S*. *aureus* (n = 4). The control rats were intraperitoneally injected with 2 ml normal saline (NS).

### Urine collection and sample preparation

Urine samples were collected from the experimental group on days 0, 1 and 2 after inoculation. Animals were individually placed in metabolic cages for 10 h to collect urine samples without any treatment (from 8 a.m to 6 p.m). After collection, the urine samples were quickly stored at -80°C. The urine samples (n = 36) were centrifuged at 12,000 g for 40 min at 4°C to remove cell debris. The supernatants were precipitated with three volumes of ethanol at -20°C overnight and then centrifuged at 12,000 g for 30 min. The pellet was resuspended in lysis buffer (8 mol/L urea, 2 mol/L thiourea, 50 mmol/L Tris, and 25 mmol/L DTT). The protein concentration of the urine samples was measured by the Bradford assay.

### Protein digestion

One hundred micrograms of urinary proteins from each sample were digested with trypsin (Trypsin Gold, Mass Spec Grade, Promega, Fitchburg, WI, USA) using filter-aided sample preparation (FASP) methods as previously described [10]. These peptide mixtures were desalted using Oasis HLB cartridges (Waters, Milford, MA) and dried by vacuum evaporation (Thermo Fisher Scientific, Bremen, Germany). The digested peptides (n = 36) were redissolved in 0.1% formic acid to a concentration of 0.5 μg/μL, and 1 μg of peptide from each sample was analyzed was for LC-MS/MS analysis in DIA mode.

### Reversed-phase fractionation spin column separation

The pooled sample was generated from equal volumes of digested peptides from each sample. A total of 90 μg of pooled peptides was separated by a high-pH reversed-phase peptide fractionation kit (Thermo Pierce, Waltham, MA, USA) according to the manufacturer’s instructions. A step gradient of increasing acetonitrile concentrations (5, 7.5, 10, 12.5, 15, 17.5, 20 and 50% acetonitrile) was added to the columns to elute peptides, and ten different fractionated samples (including the flow-through fraction, wash fraction, and eight step gradient sample fractions) of each sample were collected and dried by vacuum evaporation. The ten fractions were dried by vacuum evaporation and resuspended in 20 μl of 0.1% formic acid, and 1 μg peptide from each fraction was for LC-MS/MS analysis using the DDA mode.

### LC-MS/MS analysis

Mass spectrometry acquisition and analysis were performed using an EASY-nLC 1200 chromatography system (Thermo Fisher Scientific) and an Orbitrap Fusion Lumos Tribrid mass spectrometer (Thermo Fisher Scientific). The iRT reagent (Biognosys, Switzerland) was spiked at a concentration of 1:10 v/v into all the urinary samples for calibration of the retention time of the extracted peptide peaks. The peptide samples were loaded on a trap column (75 μm × 2 cm, 3 μm, C18, 100 Å) and a reverse-phase analysis column (75 μm × 25 cm, 2 μm, C18, 100 Å). The eluted gradient was 4%-35% buffer B (0.1% formic acid in 80% acetonitrile) at a flow rate of 400 nL/min for 90 min.

To generate the spectral library, 1 μg of each fraction from the spin column was analyzed in DDA mode. The parameters were set as follows: the full scan was acquired from 350 to 1550 m/z with a resolution of 120,000 and the MS/MS scan was performed with a resolution of 30,000 in Orbitrap; the higher-energy collisional dissociation (HCD) energy was set to 30%; the autogain control (AGC) target was set to 5.0e4; and the maximum injection time was set to 45 ms.

In DIA mode, 1 μg of each sample was analyzed. The variable isolation window of the DIA method with 36 windows was set for DIA acquisition (S1 Table). The parameters were set as follows: the full scan was acquired from 350 to 1500 m/z with a resolution of 60,000; the MS/MS scan was acquired from 200 to 2000 m/z with a resolution of 30,000; the HCD energy was set to 32%; the AGC target was set to 1.0e6; and the maximum injection time was set to 100 ms. A quality control (QC) sample of a mixture from each sample was analyzed after every six samples.

### Data analysis

The DDA data of ten fractions were processed using Proteome Discoverer software (version 2.1, Thermo Scientific) and searched against the Swiss-Prot rat database (released in 2017, including 7992 sequences) appended with the iRT peptide sequence. The search parameters were set as follows: two missed trypsin cleavage sites were allowed; the parent ion mass tolerances were set to 10 ppm; the fragment ion mass tolerances were set to 0.02 Da; the carbamidomethyl of cysteine was set as a fixed modification; and the oxidation of methionine was set as a variable modification. The false discovery rate (FDR) of proteins was less than 1%. A total of 1222 protein groups, 7554 peptide groups and 35573 peptide spectrum matches were identified. The search results were used to set the variable windows for DIA acquisition.

Ten DDA raw files were processed using Spectronaut Pulsar X (Biognosys, Switzerland) with the default parameters to generate the spectral library. Then, 36 DIA raw files of each sample were processed by using Spectronaut Pulsar X with the default setting. The results were filtered by a Q value cutoff of 0.01. The peptide intensity was based on the peak areas of the respective fragment ions for MS2, and the protein intensity was calculated by summing the intensities of their respective peptides.

### Statistical analysis

The k-nearest neighbor (K-NN) method was used to fill the missing values of protein abundance by using ’Wu Kong’ platform (https://www.omicsolution.com/wkomics/main/) [11]. The differential proteins identified on days 1 and 2 were compared with those on day 0 for each model. The differential proteins were screened by the following criteria: proteins with at least two unique peptides; fold change ≥ 1.5 or ≤ 0.67; and *P* < 0.05 in two-sided unpaired *t test*. *P-values* of groups differences were adjusted by the Bonferroni correction [12]. Grouping differences resulting in *P* < 0.05 was considered statistically significant. Orthogonal partial least squares discriminant analysis (OPLS-DA) was conducted by SIMCA software (version14.1, Umetrics, Sweden).

### Functional annotation of the differential proteins

The Database for Annotation, Visualization and Integrated Discovery (DAVID) was used to perform the functional annotation of the differential proteins and included biological processes, cellular components and molecular functions [13]. The canonical pathways were analyzed with IPA (Ingenuity Systems, Mountain View, CA, USA) software. All enriched iterms had threshold value of *P* < 0.05.

## Results

### Characterization of rats intraperitoneally injected with *E*. *coli* and *S*. *aureus*

The rats were randomly divided into the following four groups: a group (n = 4) of rats intraperitoneally injected with normal saline, a group (n = 4) of rats intraperitoneally injected with *E*. *coli* and *S*. *aureus*, a group (n = 4) of rats intraperitoneally injected with *E*. *coli*, and a group (n = 4) of rats intraperitoneally injected with *S*. *aureus*. The daily behavior changes of all the rats were observed after injection. Compared with control rats, there were no significant differences in the daily behaviors of the experimental rats.

### Urinary proteome changes

**Fig 2:**
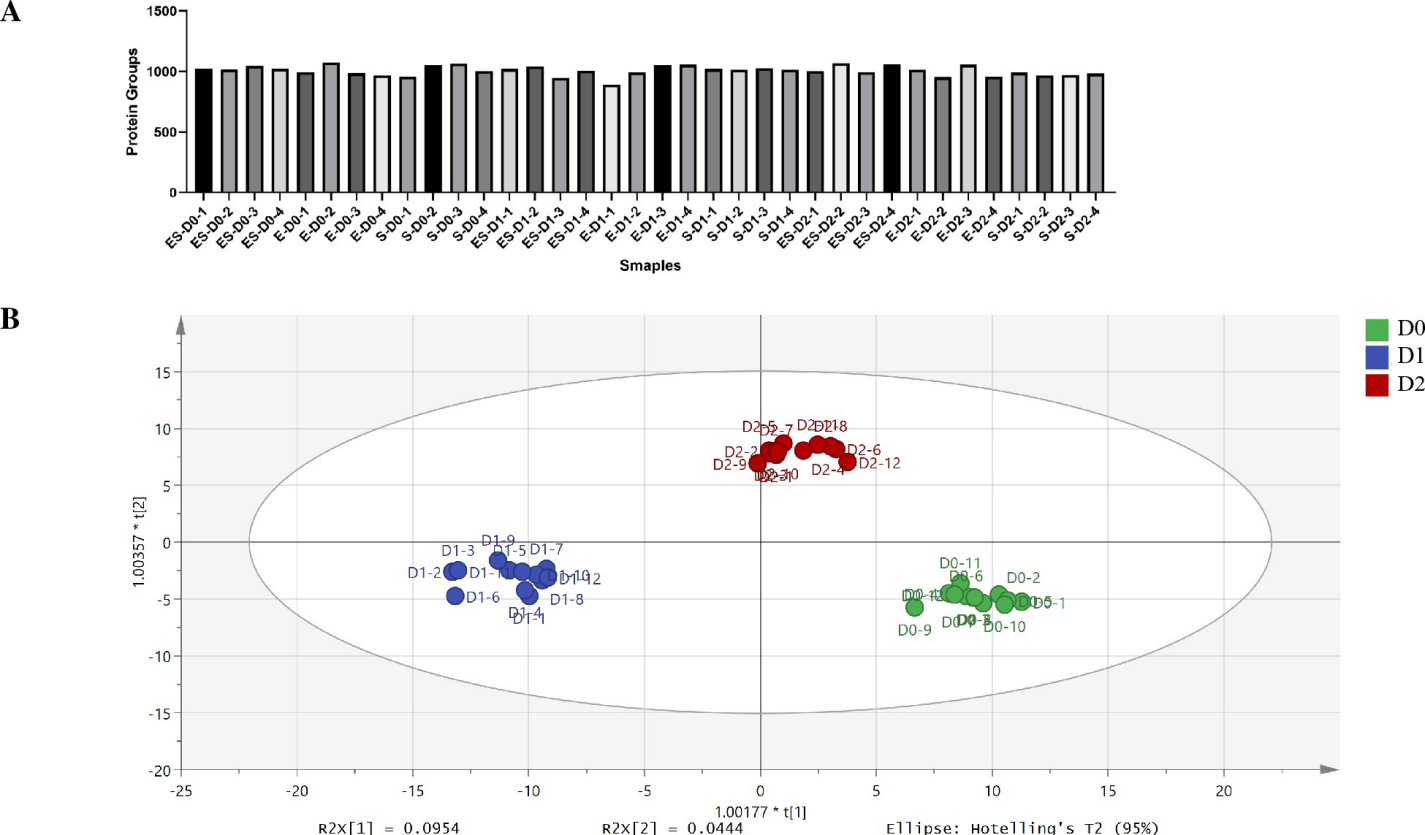
Proteomics analysis of the urine samples of three models. (A) Numbers of protein groups identified in each sample in this study. (B) OPLS-DA analysis of the 813 proteins from 36 urine samples of three models.

In this study, 36 urine samples from 12 rats at three time points (days 0, 1 and 2) were analyzed by LC-DIA-MS/MS. For DDA analysis, the spectral library contained 7849 peptides and 1254 protein groups. Then, 36 samples were analyzed by using Spectronaut X based on the spectral library. A total of 1146 protein groups were identified and the number of protein groups for each sample was presented in Fig 2A. Among them, 948 proteins with two unique peptides were for subsequent analysis. A total of 763 proteins with a coefficient of variation (CV) of the QC samples below 30% were used to fill missing value. Final, 756 high-confidence proteins were for screening differential proteins and all the identification and quantification details are listed in S2 Table.

**Fig 3:**
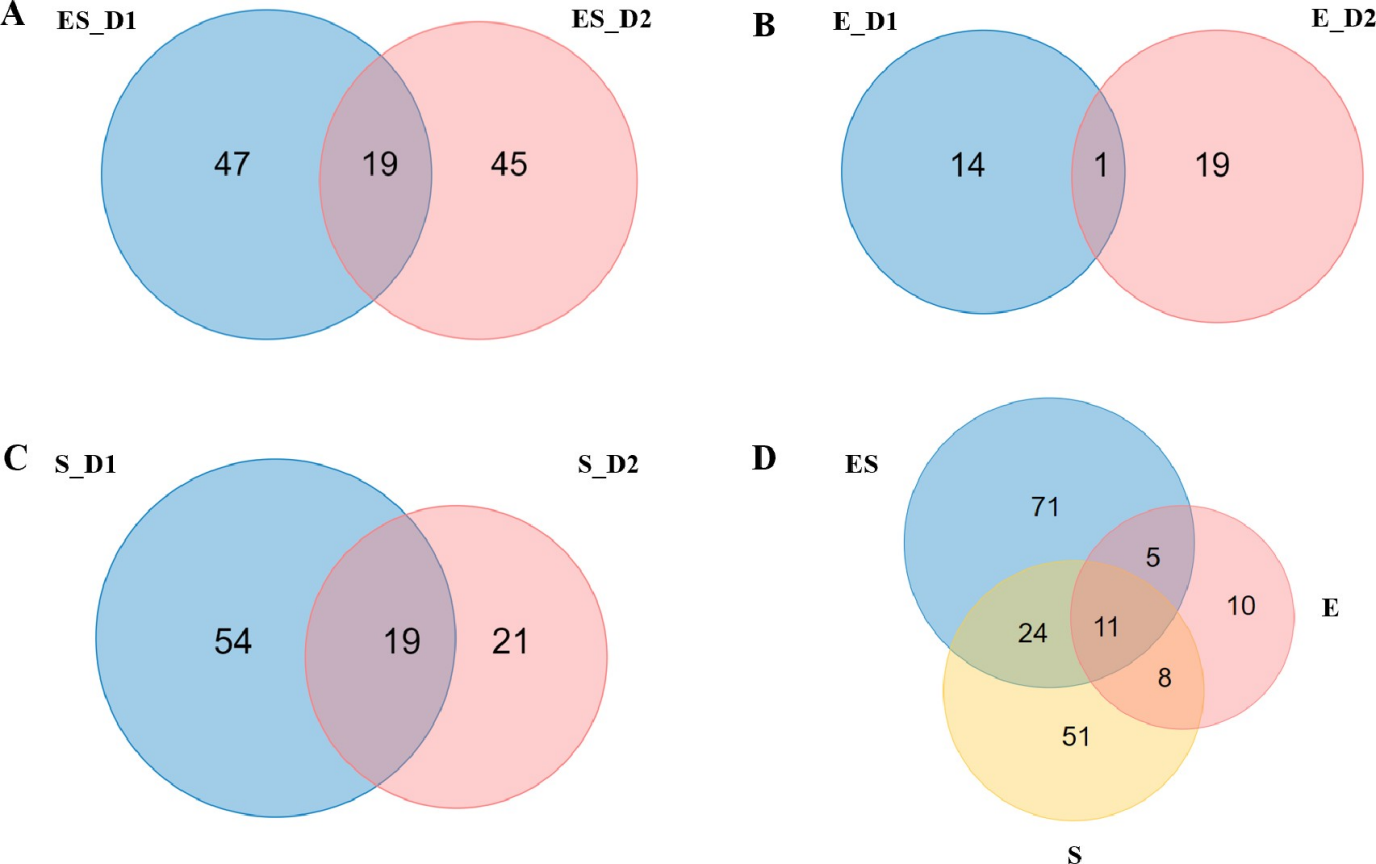
Veen diagram of differential proteins of three models. (A) Overlap evaluation of differential proteins identified at different time in ES model. (B) Overlap evaluation of differential proteins identified at different time in E model. (C) Overlap evaluation of differential proteins identified at different time in S model. (D) Overlap evaluation of differential proteins identified in three models.

First, to explore the effects of bacterial infections on the proteomic profiling, 756 proteins among three models were investigated by OPLS-DA analysis. The score plot showed that the urine samples of ES model could be separated from E model and S model, and samples at each model could be gathered together (Fig 2B). Second, the differential proteins for three models were screened by following screening criteria: fold change ≥ 1.5 or ≤ 0.67; *P* adjust < 0.05. Compared with day 0, a total of 111 proteins were significantly changed in the ES model, including 66 and 64 differential proteins on day 1 and day 2, respectively (Fig 3A, S3 Table). Compared with day 0, a total of 34 proteins were significantly changed in the E model, including 15 and 20 differential proteins on day 1 and day 2, respectively (Fig 3B, S3 Table). Compared with day 0, a total of 94 proteins were significantly changed in the S model, including 73 and 40 differential proteins on day 1 and day 2, respectively (Fig 3C, S3 Table).

### Comparative analysis of the urinary proteome

**Fig 4:**
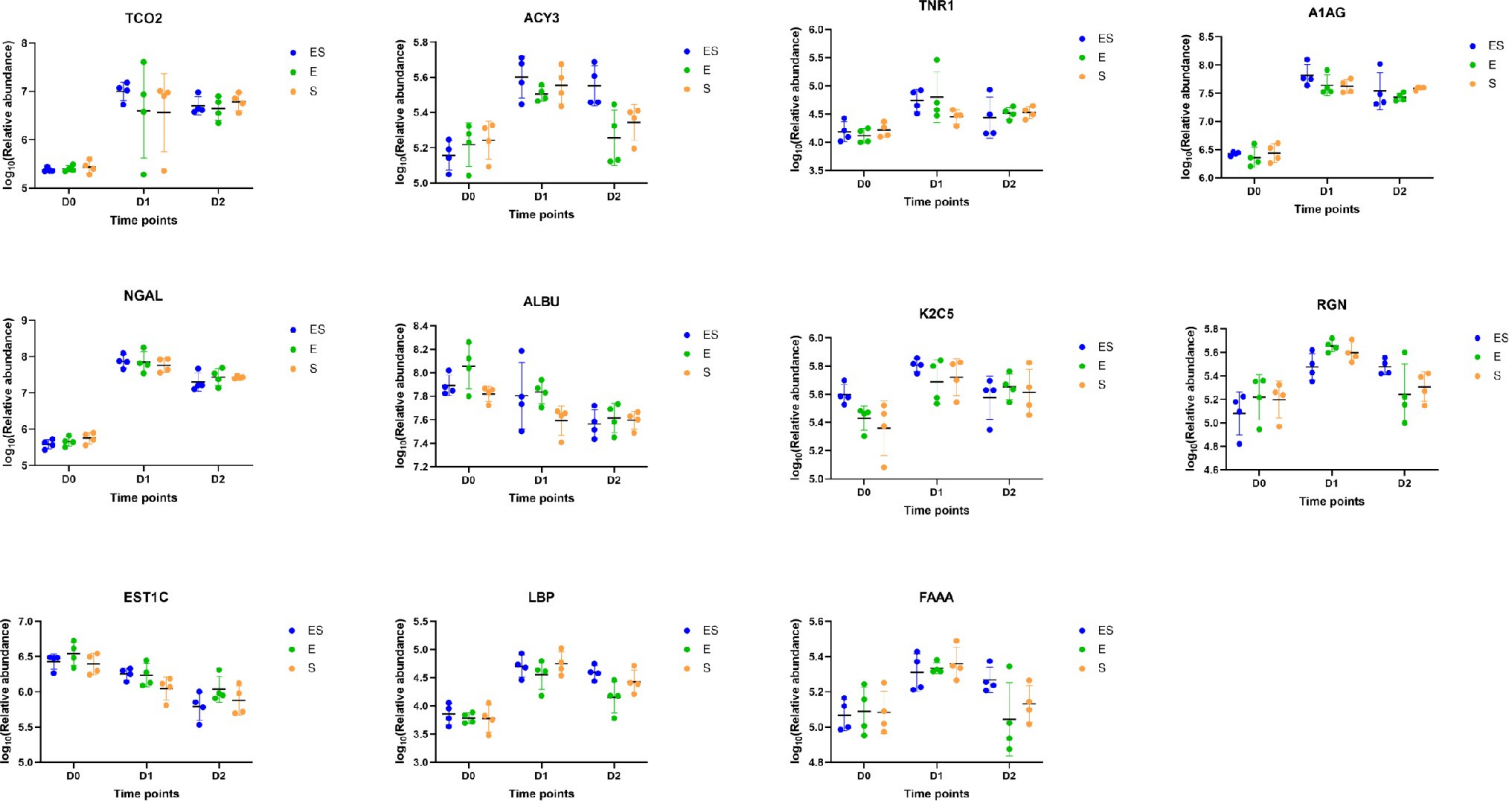
Expression of 11 differential proteins commonly identified in three models.

The overlapping differential proteins among the three models are presented in Fig 3D. Compared with the ES model, 16 (47%) differential proteins in the E model overlapped with ES model, and 35 (37%) differential proteins in the S model overlapped with ES model. Notably, 71 differential proteins were only identified in the ES model, 10 differential proteins were only identified in the E model, and 51 differential proteins were only identified in the S model. It was found that 11 differential proteins were commonly identified in the three models, among which 9 proteins showed an overall upregulated trend after bacterial infections in three models, including TCO2, ACY3, TNR1B, A1AG, NGAL, K2C5, RGN, LBP and FAAA. And 2 proteins showed a downregulated trend after bacterial infections in three models, including ALBU and EST1C (Fig 4).

### Functional comparison analysis

Functional annotation of differential proteins in the three models was conducted using DAVID and IPA software. The differential proteins in the three models were classified into biological processes, cellular components and molecular functions. A total of 111 proteins were identified in the ES model, 34 proteins were identified in the E model, and 94 proteins were identified in the S model, and all of the proteins were annotated. All the representative iterms were considered to be significant at *P* < 0.05.

**Fig 5:**
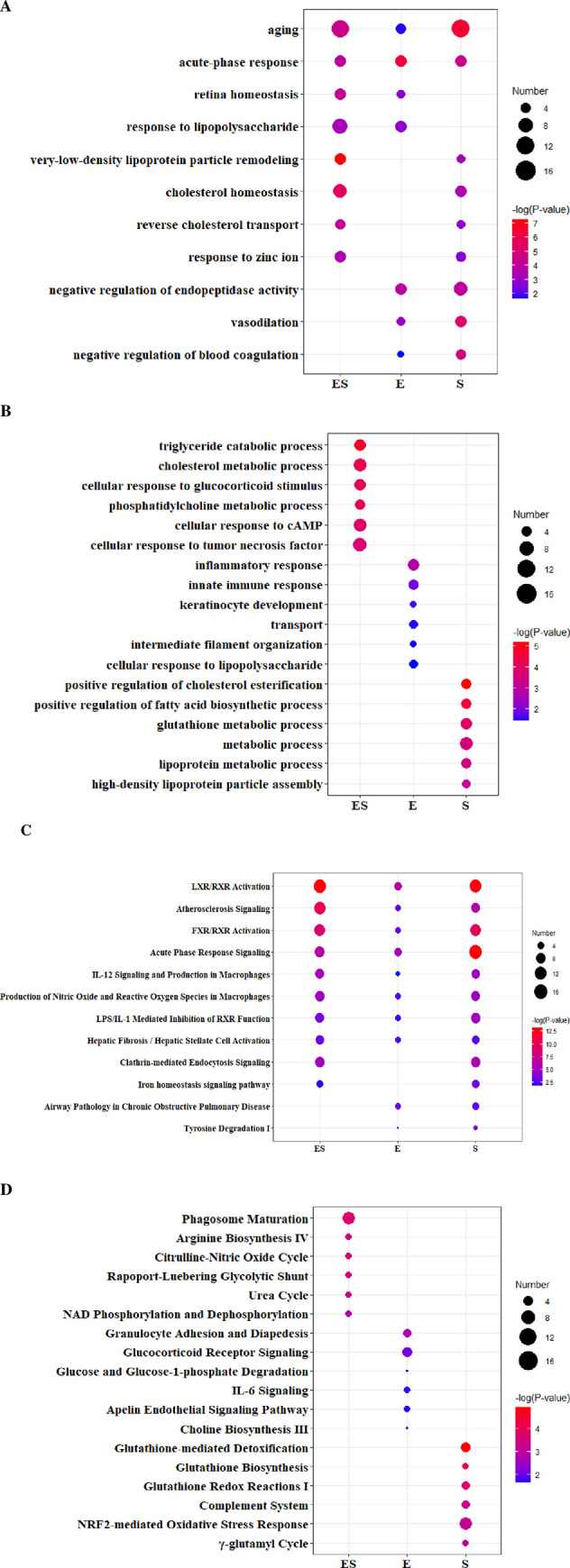
Functional enrichment analysis of differential proteins of three models. (A) Shared biological processes in three models. (B) Unique biological processes in three models. (C) Shared canonical pathways in three models. (D) Unique canonical pathways in three models.

In the biological process category, a total of 68, 13, and 63 iterms were overrepresented in the ES model, E model, and S model, respectively, and 6 items were commonly enriched among the three models (S4 Table). Notably, 32 unique iterms were enriched in the ES model, and 2 and 24 unique iterms were enriched in the E and S models, respectively. As shown in Fig 5A, several functional iterms were shared in three models. For example, aging and acute-phase response were enriched in three models; retina homeostasis and response to lipopolysaccharide were enriched in ES and E model; very-low-density lipoprotein particle remodeling and cholesterol homeostasis were enriched in ES and S model; negative regulation of endopeptidase activity, vasodilation, and negative regulation of blood coagulation were enriched in E and S model. In addition, some functional iterms were only enriched in ES model (Fig 5B), such as triglyceride catabolic process, cholesterol metabolic process, cellular response to glucocorticoid stimulus, and phosphatidylcholine metabolic process. Some functional iterms were only enriched in E model (Fig 5B), such as inflammatory response and innate immune response. Some functional iterms were only enriched in S model (Fig 5B), such as positive regulation of cholesterol esterification, positive regulation of fatty acid biosynthetic process, and glutathione metabolic process.

By IPA analysis, it was found that the differential proteins were involved in some major biological pathways. A total of 42, 27, and 49 iterms were overrepresented in the ES model, E model and S model, respectively, and 13 items were commonly enriched among the three models (S4 Table). Notably, 20 unique iterms were only enriched in the ES model, and 12 and 27 unique iterms were only enriched in the E model and S model, respectively. As shown in Fig 5C, several pathways were enriched among all three models, such as LXR/RXR activation, atherosclerosis signaling, FXR/RXR activation, acute phase response signaling, IL-12 signaling and production in macrophages, production of nitric oxide and reactive oxygen species in macrophages, and LPS/IL-1 mediated inhibition of RXR function. In addition, some pathways were only enriched in the ES model (Fig 5D), such as phagosome maturation, arginine biosynthesis IV, and citrulline-nitric oxide cycle. Some pathways were only enriched in the E model (Fig 5D), such as granulocyte adhesion and diapedesis, glucocorticoid receptor signaling, glucose and glucose-1-phosphate degradation. Some pathways were only enriched in the S model (Fig 5D), such as glutathione biosynthesis, glutathione redox reactions I, and complement system.

In the cellular component category, the differential proteins of the three models mainly came from extracellular exosome, extracellular space, blood microparticle, and extracellular region (S1 Fig). In the molecular function category, cholesterol transporter activity, fatty acid binding, drug binding, and phosphatidylcholine-sterol O-acyltransferase activator activity were overrepresented in the ES and S model. Procollagen-lysine 5-dioxygenase activity and acid phosphatase activity were only overrepresented in the ES model. Endopeptidase inhibitor activity and structural molecule activity were only overrepresented in the ES model. Glutathione binding, glutathione transferase activity, and cholesterol transporter activity were only overrepresented in the S model (S2 Fig).

## Discussion

In this study, two bacterial single injection and co-injection rat models were established by intraperitoneal injection of *E*. *coli* and *S*. *aureus*. Urinary proteomes on days 0, 1 and 2 of the three models were analyzed by LC-MS/MS. A total of 111, 34 and 94 differentially expressed proteins were identified in the ES model, E model and S model, respectively.

Among them, some differential proteins were reported to be associated with bacterial infection. For example, lipopolysaccharide-binding protein (LBP) was indispensable for the induction of innate immune response to small amounts of Gram-negative bacteria [14]. Phosphoglycerate mutase (PGAM1) was reported to be upregulated in *Staphylococcus aureus* biofilm formation [15]. Argininosuccinate synthase (ASSY) plays a role in the innate immune response to bacterial infections [16]. Galectin-3-binding protein (LG3BP) is a glycoprotein with innate immune function in viral and bacterial infections [17]. Studies have shown that increased concentrations of metalloproteinase inhibitor 1 (TIMP1) in cerebrospinal fluid were part of the host response to bacterial meningitis [18]. Pleural effusion adenosine deaminase (ADA)can be used to distinguish between Gram-negative and Gram-positive infections of the pleural cavity early and quickly [19]. Complement C4 (CO4) is a key molecule in the complement system and one of the main components of innate immunity, which can immediately identify and eliminate invading microorganisms [20]. Apolipoprotein E (APOE) was a novel diagnostic marker for invasive bacterial infections in pediatric patients [21]. Eosinophil cationic protein (ECP) is a protein secreted by eosinophils, which has high inhibitory activity against both gram-negative bacteria and gram-positive bacteria [22]. CD9 antigen (CD9) plays a key role in bacterial adhesion [23]. It was reported that complement component C9 (CO9) could enhance the capacity of beta-lactam antibiotics to kill *Escherichia coli* [24]. These results showed that the urinary proteome could reflect changes of bacterial injection.

Approximately 47% differential proteins in the E model overlapped with ES model, and 36% differential proteins in the S model overlapped with ES model. Some inflammatory processes were enriched in all three models. Compared with E and S model, 71 unique differential proteins were identified in the ES model. Additionally, the differential proteins were involved in several unique biological processes. These results indicated that the differential proteins identified in the two bacteria co-injection rat model were not simple superposition of differential proteins identified in the single bacteria injection rat models. Our study will help to better understand the complexity of the urinary proteome of multifactor diseases.

The urine proteomes of the three models were different even after the same numbers of two bacteria were injected intraperitoneally. Interestingly, the largest number of differential urinary proteins was identified in the ES model, which suggested that the number of differential proteins may be related to complexity. The number of unique differential proteins identified in the ES model was also the largest, which may be due to more differences produced by the co-injection than by the single injection.

In the future of precision medicine, a larger number of samples are needed to establish databases for complex diseases due to individual differences. The self-controlled method is suitable for clinically studying urine biomarker discovery to reduce the interference of various factors. This is a preliminary study on the effects of co-injection with two bacteria and single injection on urinary proteins in animal models with the limited samples. The relationship between single and multifactor diseases on the urinary proteome will be elaborated with the help of artificial intelligence in a larger number of clinical samples.

## Conclusions

Our results indicated that (1) the urine proteome could distinguish different bacterial intraperitoneal injections models and (2) the effects of co-injection with two bacteria on the urine proteome were not simple superposition of single bacteria injection.

## Supporting information

S1 Fig (PNG)

S2 Fig (TIF)

S1 Table The variable isolation window of the DIA method with 36 windows was set for DIA acquisition. (XLSX)

S2 Table The identification and quantification details of 813 proteins identified in this study. (XLSX)

S3 Table The differential proteins of three models. (XLSX)

S4 Table The terms of biological process and pathways of three models. (XLSX)
