# Supplementary figures and images for: Comparison of urine proteome among rat models by intraperitoneal injection with single bacteria and co-injection with two bacteria
Source: PLoS One. 2021 Dec 31;16(12):e0261488. doi: 10.1371/journal.pone.0261488 (PMC8719777; doi:10.1371/journal.pone.0261488)

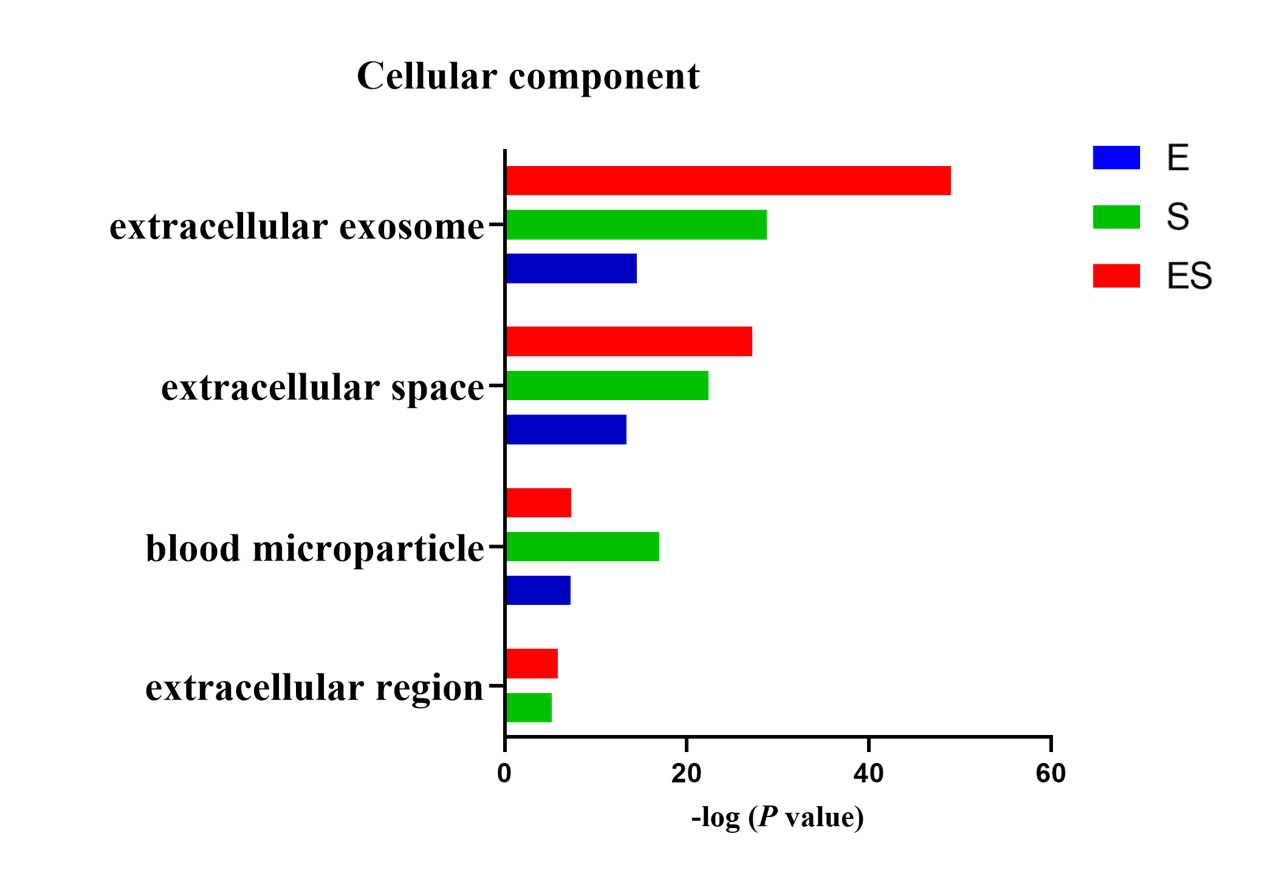

Supplement: S1 Fig — (PNG) [file pone.0261488.s001.png]

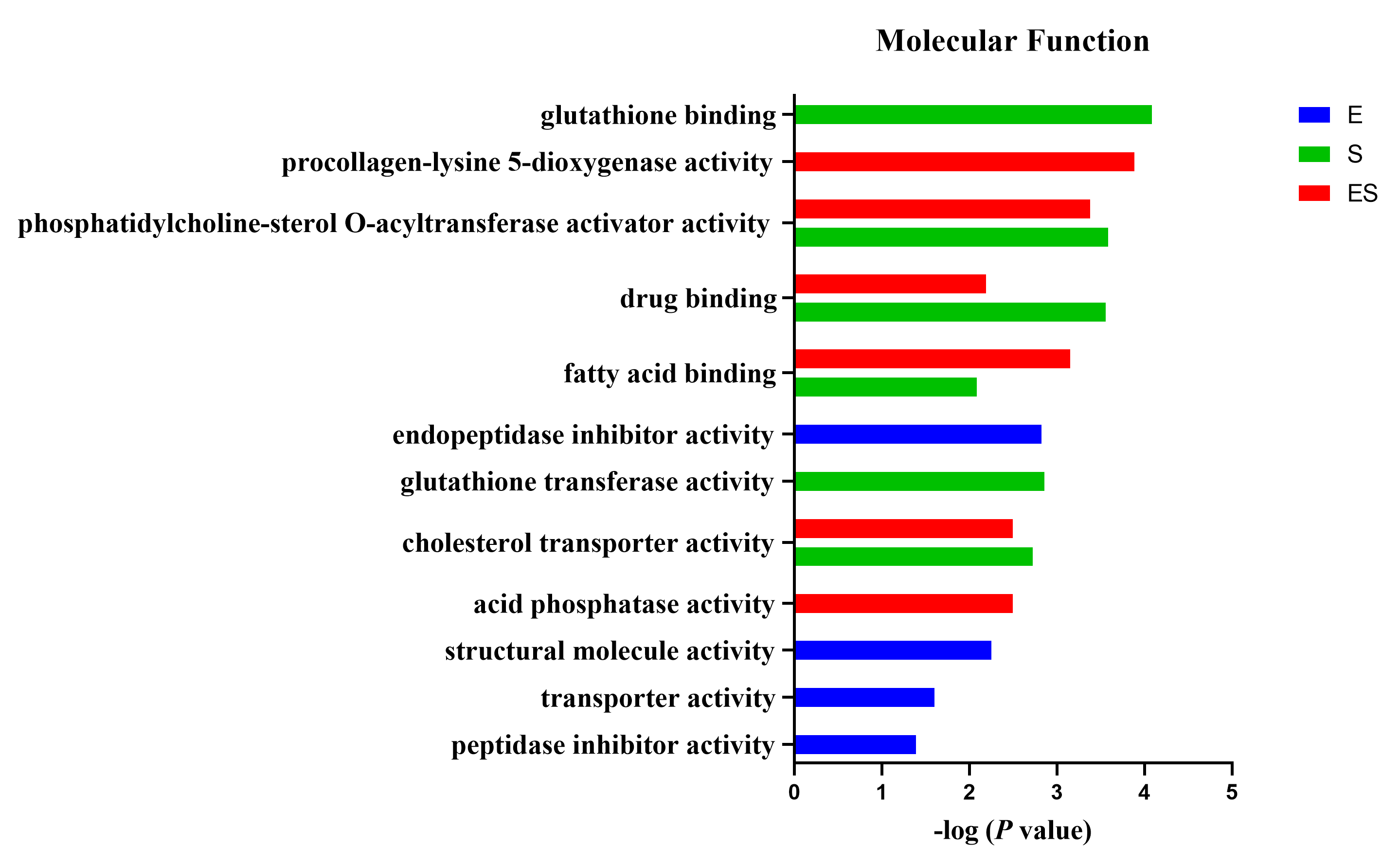

Supplement: S2 Fig — (TIF) [file pone.0261488.s002.tif]
